# 6-De­oxy-3,4-*O*-isopropyl­idene-2-*C*-methyl-l-galactono-1,5-lactone

**DOI:** 10.1107/S1600536811034957

**Published:** 2011-08-31

**Authors:** Sarah F. Jenkinson, Loren L. Parry, Francis X. Wilson, George W. J. Fleet, David J. Watkin

**Affiliations:** a Department of Organic Chemistry, Chemistry Research Laboratory, University of Oxford, Oxford OX1 3TA, England; b Summit PLC, 91 Milton Park, Abingdon, Oxfordshire OX14 4RY, England; c Department of Chemical Crystallography, Chemistry Research Laboratory, University of Oxford, Oxford OX1 3TA, England

## Abstract

X-ray crystallography unequivocally confirmed the stereochemistry of the 2-*C*-methyl group in the title mol­ecule, C_10_H_16_O_5_, in which the 1,5-lactone ring exists in a boat conformation. The absolute stereochemistry was determined by the use of d-ribose in the synthesis. The crystal exists as O—H⋯O hydrogen bonded chains of mol­ecules running parallel to the *a* axis with each mol­ecule acting as a donor and acceptor for one hydrogen bond.

## Related literature

For branched imino­sugars, see: Håkansson *et al.* (2007 ▶, 2008 ▶); Asano *et al.* (2000 ▶); da Cruz *et al.* (2011 ▶); Best *et al.* (2010 ▶) and for branched sugars, see: Booth *et al.* (2008 ▶, 2009 ▶); da Cruz *et al.* (2008 ▶); Hotchkiss *et al.* (2006 ▶, 2007 ▶); Jenkinson *et al.* (2007 ▶); Jones *et al.* (2007 ▶, 2008 ▶); Rao *et al.* (2008 ▶). For conformations of related 1,5-lactones, see: Baird *et al.* (1987 ▶); Booth *et al.* (2007*a* ▶,*b* ▶); Bruce *et al.* (1990 ▶); Punzo *et al.* (2005 ▶, 2006 ▶); Dai *et al.* (2010 ▶).
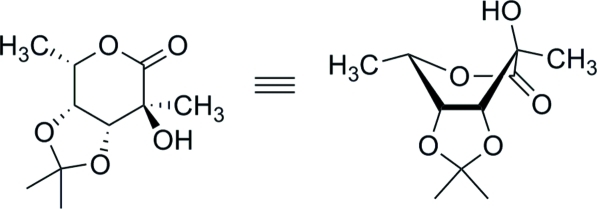


## Experimental

### 

#### Crystal data

C_10_H_16_O_5_
*M* *_r_* = 216.23
Orthorhombic, 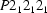

*a* = 6.1132 (2) Å
*b* = 12.2963 (4) Å
*c* = 14.6367 (5) Å
*V* = 1100.24 (6) Å^3^
*Z* = 4
Mo *K*α radiation
μ = 0.11 mm^−1^
*T* = 150 K
0.20 × 0.20 × 0.04 mm

#### Data collection

Nonius KappaCCD diffractometer
Absorption correction: multi-scan (*DENZO*/*SCALEPACK*; Otwinowski & Minor, 1997 ▶) *T*_min_ = 0.95, *T*_max_ = 1.00
8628 measured reflections
1454 independent reflections
1131 reflections with *I* > 2σ(*I*)
*R*_int_ = 0.067

#### Refinement

*R*[*F* ^2^ > 2σ(*F* ^2^)] = 0.038
*wR*(*F* ^2^) = 0.090
*S* = 0.89
1454 reflections
136 parameters
H-atom parameters constrained
Δρ_max_ = 0.40 e Å^−3^
Δρ_min_ = −0.29 e Å^−3^

### 

Data collection: *COLLECT* (Nonius, 2001 ▶); cell refinement: *DENZO*/*SCALEPACK* (Otwinowski & Minor, 1997 ▶); data reduction: *DENZO*/*SCALEPACK*; program(s) used to solve structure: *SIR92* (Altomare *et al.*, 1994 ▶); program(s) used to refine structure: *CRYSTALS* (Betteridge *et al.*, 2003 ▶); molecular graphics: *CAMERON* (Watkin *et al.*, 1996 ▶); software used to prepare material for publication: *CRYSTALS*.

## Supplementary Material

Crystal structure: contains datablock(s) global, I. DOI: 10.1107/S1600536811034957/lh5321sup1.cif

Structure factors: contains datablock(s) I. DOI: 10.1107/S1600536811034957/lh5321Isup2.hkl

Additional supplementary materials:  crystallographic information; 3D view; checkCIF report

## Figures and Tables

**Table 1:** Hydrogen-bond geometry (Å, °)

*D*—H⋯*A*	*D*—H	H⋯*A*	*D*⋯*A*	*D*—H⋯*A*
O11—H111⋯O1^i^	0.86	1.93	2.793 (3)	172

Symmetry code: (i) 
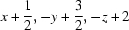
.

**Fig. 1.**
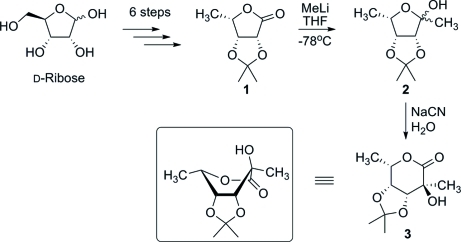
Synthetic Scheme.

**Fig. 2.**
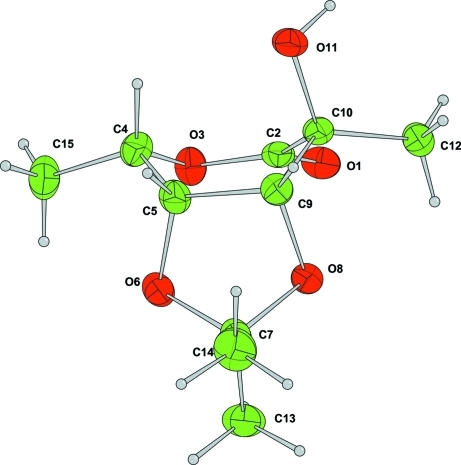
The title compound with displacement ellipsoids drawn at the 50% probability level. H atoms are shown as spheres of arbitary radius.

**Fig. 3.**
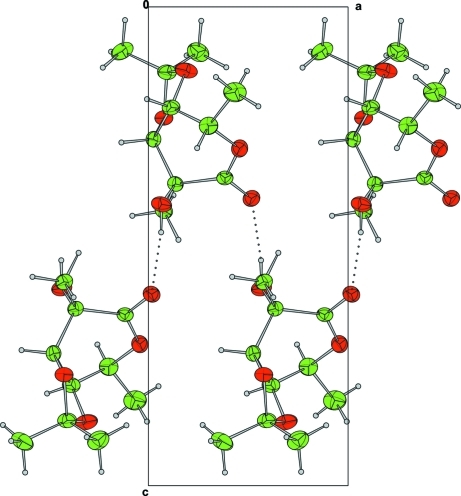
Packing diagram of the title compound projected along the b-axis. Hydrogen bonds are shown by dotted lines.

## supplementary crystallographic information

### Comment

2-*C*-Methyl branched sugars, as well as being chirons for the enantiospecific synthesis of complex targets (Hotchkiss *et al.*, 2006; Hotchkiss *et al.*, 2007; da Cruz *et al.*, 2008; Booth *et al.*, 2009) including 2'-*C*-methyl nucleosides (Jenkinson *et al.*, 2007), are a class of rare sugars with chemotherapeutic potential (Rao *et al.*, 2008; Jones *et al.*, 2008; Booth *et al.*, 2008). Branched iminosugars have also been shown to exhibit interesting biological activity. For example: 6-*C*-methyl-swainsonine is a more potent inhibitor of *L*-rhamnosidase than *L*-swainsonine (Håkansson *et al.*, 2007; Håkansson *et al.*, 2008; Asano *et al.*, 2000); 4-*C*-methylDAB and 4-*C*-methylLAB are both potent and specific α-glucosidase inhibitors (da Cruz *et al.* 2011) and isoLAB has been shown to partially rescue the defective F508del-CFTR function and therefore may have a role in the study of cyctic fibrosis (Best *et al.*, 2010).

D-ribose 1 was converted by a number of steps to the lactols 2 (Fig. 1). The reaction of 2 with sodium cyanide in water gave a chain extension to afford a single isolated crystalline product 3 (Fig. 2).

3,4-*O*-Isopropylidene-1,5-lactones, such as 3, invariably crystallize in boat conformations (Baird *et al.*, 1987; Bruce *et al.*, 1990; Punzo *et al.*, 2005). The diastereoselectivity of the reaction may be rationalized by the formation of the lactone 3 with less steric congestion (Punzo *et al.*, 2006; Booth *et al.*, 2007*a*; Booth *et al.*, 2007*b*; Dai *et al.* 2010) with the smaller hydroxy group rather than the methyl group in the flagpole position (Fig. 2). The structure of 3 was confirmed by the X-ray crystallographic analysis. The absolute configuration was assigned from the use of D-ribose as the starting material. The title compound exists as O—H···O hydrogen bonded chains of molecules running parallel to the *a*-axis (Fig. 3). Each molecule acts as a donor and acceptor for 1 hydrogen bond. Only classical hydrogen bonding has been considered.

### Experimental

The title compound was recrystallized by diffusion from a mixture of ethyl acetate and cyclohexane: m.p. 369–371 K; [α]_D_^25^ -84.6 (*c*, 1.03 in CHCl_3_).

### Refinement

In the absence of significant anomalous scattering, Friedel pairs were merged and the absolute configuration was assigned from the use of D-ribose as the starting material.

The H atoms were all located in a difference map, but those attached to carbon atoms were repositioned geometrically. The H atoms were initially refined with soft restraints on the bond lengths and angles to regularize their geometry (C—H in the range 0.93–0.98, O—H = 0.82 Å) and *U*_iso_(H) (in the range 1.2–1.5 times*U*_eq_ of the parent atom), after which the positions were refined with riding constraints.

### Crystal data

**Table:**

C_10_H_16_O_5_	*F*(000) = 464
*M**_r_* = 216.23	*D*_x_ = 1.305 Mg m^−^^3^
Orthorhombic, *P*2_1_2_1_2_1_	Mo *K*α radiation, λ = 0.71073 Å
Hall symbol: P 2ac 2ab	Cell parameters from 1433 reflections
*a* = 6.1132 (2) Å	θ = 5–27°
*b* = 12.2963 (4) Å	µ = 0.11 mm^−^^1^
*c* = 14.6367 (5) Å	*T* = 150 K
*V* = 1100.24 (6) Å^3^	Plate, colourless
*Z* = 4	0.20 × 0.20 × 0.04 mm

### Data collection

**Table:**

Nonius KappaCCD diffractometer	1131 reflections with *I* > 2σ(*I*)
graphite	*R*_int_ = 0.067
ω scans	θ_max_ = 27.5°, θ_min_ = 5.2°
Absorption correction: multi-scan (*DENZO*/*SCALEPACK*; Otwinowski & Minor, 1997)	*h* = −7→7
*T*_min_ = 0.95, *T*_max_ = 1.00	*k* = −15→15
8628 measured reflections	*l* = −18→19
1454 independent reflections	

### Refinement

**Table:**

Refinement on *F*^2^	Primary atom site location: structure-invariant direct methods
Least-squares matrix: full	Hydrogen site location: inferred from neighbouring sites
*R*[*F*^2^ > 2σ(*F*^2^)] = 0.038	H-atom parameters constrained
*wR*(*F*^2^) = 0.090	Method = Modified Sheldrick *w* = 1/[σ^2^(*F*^2^) + (0.06*P*)^2^ + 0.14*P*], where *P* = [max(*F*_o_^2^,0) + 2*F*_c_^2^]/3
*S* = 0.89	(Δ/σ)_max_ = 0.000259
1454 reflections	Δρ_max_ = 0.40 e Å^−^^3^
136 parameters	Δρ_min_ = −0.29 e Å^−^^3^
0 restraints	

### Fractional atomic coordinates and isotropic or equivalent isotropic displacement parameters (Å^2^)

**Table:**

	*x*	*y*	*z*	*U*_iso_*/*U*_eq_	
O1	−0.0165 (3)	0.80556 (12)	0.89995 (10)	0.0290	
C2	0.1164 (4)	0.75275 (16)	0.85712 (12)	0.0226	
O3	0.0426 (3)	0.68163 (11)	0.79561 (10)	0.0287	
C4	0.1986 (4)	0.60986 (16)	0.74843 (14)	0.0289	
C5	0.3861 (4)	0.67578 (16)	0.71001 (14)	0.0271	
O6	0.3108 (3)	0.73556 (12)	0.63264 (9)	0.0355	
C7	0.4068 (4)	0.84132 (17)	0.63637 (13)	0.0292	
O8	0.4222 (3)	0.86365 (10)	0.73198 (9)	0.0261	
C9	0.4744 (4)	0.76299 (15)	0.77626 (13)	0.0240	
C10	0.3643 (4)	0.76166 (16)	0.86972 (13)	0.0221	
O11	0.4337 (3)	0.66259 (11)	0.91228 (10)	0.0301	
C12	0.4222 (4)	0.86099 (16)	0.92581 (13)	0.0277	
C13	0.2506 (5)	0.92177 (19)	0.59465 (16)	0.0401	
C14	0.6306 (5)	0.8415 (2)	0.59246 (16)	0.0422	
C15	0.0664 (5)	0.55020 (19)	0.67809 (16)	0.0402	
H41	0.2554	0.5572	0.7948	0.0338*	
H51	0.5068	0.6260	0.6926	0.0337*	
H91	0.6348	0.7543	0.7832	0.0290*	
H121	0.5803	0.8627	0.9363	0.0432*	
H122	0.3490	0.8570	0.9864	0.0426*	
H123	0.3742	0.9272	0.8933	0.0431*	
H131	0.3082	0.9961	0.6014	0.0583*	
H132	0.2349	0.9048	0.5289	0.0582*	
H133	0.1099	0.9171	0.6262	0.0586*	
H141	0.6868	0.9161	0.5934	0.0627*	
H142	0.7280	0.7929	0.6268	0.0622*	
H143	0.6166	0.8179	0.5275	0.0623*	
H153	0.1616	0.4999	0.6465	0.0610*	
H152	−0.0508	0.5092	0.7077	0.0613*	
H151	0.0062	0.6022	0.6340	0.0612*	
H111	0.4361	0.6716	0.9708	0.0460*	

### Atomic displacement parameters (Å^2^)

**Table:**

	*U*^11^	*U*^22^	*U*^33^	*U*^12^	*U*^13^	*U*^23^
O1	0.0284 (9)	0.0329 (8)	0.0257 (7)	0.0061 (7)	0.0025 (7)	0.0035 (7)
C2	0.0264 (11)	0.0224 (10)	0.0191 (8)	0.0006 (10)	0.0006 (9)	0.0049 (9)
O3	0.0263 (8)	0.0302 (8)	0.0295 (8)	−0.0029 (7)	0.0011 (6)	−0.0051 (6)
C4	0.0344 (13)	0.0224 (10)	0.0299 (11)	0.0027 (9)	0.0008 (10)	−0.0023 (9)
C5	0.0315 (12)	0.0276 (10)	0.0222 (9)	0.0034 (10)	0.0012 (10)	−0.0035 (9)
O6	0.0500 (11)	0.0332 (8)	0.0234 (7)	−0.0145 (8)	−0.0068 (7)	0.0026 (7)
C7	0.0354 (13)	0.0305 (12)	0.0216 (10)	−0.0092 (11)	−0.0011 (10)	−0.0005 (9)
O8	0.0326 (8)	0.0250 (7)	0.0208 (7)	−0.0002 (7)	0.0006 (6)	0.0007 (6)
C9	0.0220 (11)	0.0253 (10)	0.0248 (9)	0.0032 (9)	0.0000 (9)	−0.0009 (9)
C10	0.0246 (11)	0.0198 (10)	0.0220 (9)	0.0042 (9)	−0.0017 (9)	0.0009 (8)
O11	0.0405 (9)	0.0267 (7)	0.0233 (7)	0.0089 (7)	−0.0047 (7)	0.0023 (6)
C12	0.0300 (12)	0.0288 (11)	0.0243 (10)	0.0005 (10)	−0.0028 (10)	−0.0022 (9)
C13	0.0453 (16)	0.0394 (13)	0.0355 (12)	−0.0026 (13)	−0.0080 (13)	0.0110 (12)
C14	0.0470 (16)	0.0480 (14)	0.0316 (11)	−0.0057 (14)	0.0137 (12)	−0.0004 (12)
C15	0.0509 (17)	0.0330 (12)	0.0368 (12)	−0.0104 (12)	−0.0011 (13)	−0.0088 (10)

### Geometric parameters (Å, °)

**Table:**

O1—C2	1.214 (2)	C9—H91	0.992
C2—O3	1.334 (2)	C10—O11	1.433 (2)
C2—C10	1.530 (3)	C10—C12	1.514 (3)
O3—C4	1.472 (3)	O11—H111	0.864
C4—C5	1.512 (3)	C12—H121	0.979
C4—C15	1.501 (3)	C12—H122	0.995
C4—H41	1.000	C12—H123	0.987
C5—O6	1.426 (2)	C13—H131	0.985
C5—C9	1.543 (3)	C13—H132	0.989
C5—H51	0.992	C13—H133	0.978
O6—C7	1.428 (3)	C14—H141	0.979
C7—O8	1.429 (2)	C14—H142	0.982
C7—C13	1.505 (3)	C14—H143	0.997
C7—C14	1.512 (3)	C15—H153	0.967
O8—C9	1.433 (2)	C15—H152	0.977
C9—C10	1.525 (3)	C15—H151	0.980
			
O1—C2—O3	118.2 (2)	C2—C10—O11	106.55 (17)
O1—C2—C10	124.18 (18)	C9—C10—O11	105.58 (16)
O3—C2—C10	117.60 (18)	C2—C10—C12	110.78 (18)
C2—O3—C4	119.38 (17)	C9—C10—C12	112.01 (17)
O3—C4—C5	110.13 (15)	O11—C10—C12	112.40 (15)
O3—C4—C15	105.41 (19)	C10—O11—H111	109.1
C5—C4—C15	114.54 (18)	C10—C12—H121	109.5
O3—C4—H41	107.1	C10—C12—H122	109.8
C5—C4—H41	109.7	H121—C12—H122	107.8
C15—C4—H41	109.6	C10—C12—H123	109.5
C4—C5—O6	109.09 (18)	H121—C12—H123	110.5
C4—C5—C9	113.83 (17)	H122—C12—H123	109.7
O6—C5—C9	104.69 (15)	C7—C13—H131	110.0
C4—C5—H51	109.2	C7—C13—H132	108.5
O6—C5—H51	110.7	H131—C13—H132	109.2
C9—C5—H51	109.3	C7—C13—H133	109.2
C5—O6—C7	107.85 (16)	H131—C13—H133	108.7
O6—C7—O8	103.85 (15)	H132—C13—H133	111.2
O6—C7—C13	108.81 (18)	C7—C14—H141	108.2
O8—C7—C13	108.23 (18)	C7—C14—H142	109.3
O6—C7—C14	110.92 (18)	H141—C14—H142	110.5
O8—C7—C14	110.89 (19)	C7—C14—H143	109.1
C13—C7—C14	113.65 (18)	H141—C14—H143	108.4
C7—O8—C9	106.95 (14)	H142—C14—H143	111.3
C5—C9—O8	103.77 (15)	C4—C15—H153	108.4
C5—C9—C10	113.70 (17)	C4—C15—H152	110.1
O8—C9—C10	108.47 (15)	H153—C15—H152	108.9
C5—C9—H91	109.6	C4—C15—H151	109.6
O8—C9—H91	111.1	H153—C15—H151	109.1
C10—C9—H91	110.1	H152—C15—H151	110.7
C2—C10—C9	109.25 (16)		

### Hydrogen-bond geometry (Å, °)

**Table:**

*D*—H···*A*	*D*—H	H···*A*	*D*···*A*	*D*—H···*A*
C13—H131···O11^i^	0.99	2.59	3.536 (3)	161
O11—H111···O1^ii^	0.86	1.93	2.793 (3)	172

Symmetry codes: (i) −*x*+1, *y*+1/2, −*z*+3/2; (ii) *x*+1/2, −*y*+3/2, −*z*+2.
